# Self-Rated Health as a Reliable Predictor of Incident Frailty in the Presence of Competing Mortality

**DOI:** 10.1093/geroni/igaf122.234

**Published:** 2025-12-31

**Authors:** Mallak Alzahrani, Megan Huisingh-Scheetz, Melissa Hladek, Qian-Li Xue

**Affiliations:** Johns Hopkins University, Baltimore, Maryland, United States; The University of Chicago, Chicago, Illinois, United States; Johns Hopkins University, Baltimore, Maryland, United States; Johns Hopkins University, Baltimore, Maryland, United States

## Abstract

Self-rated health (SRH) predicts frailty in longitudinal studies, but failing to account for competing mortality risks may bias its predictive value for frailty. Using data from 4,755 adults aged 65+ who participated in the National Health and Aging Trends Study between 2011 and 2019, we analyzed the association between SRH and incident frailty while accounting for frailty-free mortality (i.e., death before frailty onset). Included in the analysis are those who lived in the community or residential care settings, had no probable dementia, and were not frail (per the physical frailty phenotype) at baseline. Over a median follow-up of 4 years, 1,564 developed frailty (74 per 1000 person-years), and 639 died before frailty onset (30 per 1000 person-years). After adjusting for demographics, number of chronic diseases, and psychological wellbeing, worse SRH independently predicted a higher risk of both frailty and frailty-free mortality, with a stronger association for frailty. Specifically, compared to those reporting excellent health, participants rating their health as very good, good, fair, and poor respectively had 1.3, 1.8, 2.6, 3.3 times higher frailty risk (all p < 0.05), and 1.2, 1.5, 1.4, 2.5 times higher risk of frailty-free mortality (p = 0.11, 0.01, 0.05, < 0.01). The associations remained strong across age groups. These findings suggest that older adults may intuitively perceive subtle functional declines before clinical frailty emerges. The weaker link to frailty-free mortality may reflect the role of unpredictable acute health events. As a low-cost, scalable tool, SRH can help identify early frailty risk and guide targeted prevention efforts.

